# Perceived emotional intelligence as a moderator variable between cybervictimization and its emotional impact

**DOI:** 10.3389/fpsyg.2015.00486

**Published:** 2015-04-23

**Authors:** Paz Elipe, Joaquín A. Mora-Merchán, Rosario Ortega-Ruiz, José A. Casas

**Affiliations:** ^1^Department of Psychology, Faculty of Humanities and Education, University of JaénJaén, Spain; ^2^Department of Developmental and Educational Psychology, University of SevilleSeville, Spain; ^3^Faculty of Psychology, University of CórdobaCórdoba, Spain

**Keywords:** perceived emotional intelligence, cyberbullying, cybervictimization, emotional impact, emotions

## Abstract

The negative effects of traditional bullying and, recently, cyberbullying on victims are well-documented, and abundant empirical evidence for it exists. Cybervictimization affects areas such as academic performance, social integration and self-esteem, and causes emotions ranging from anger and sadness to more complex problems such as depression. However, not all victims are equally affected, and the differences seem to be due to certain situational and personal characteristics. The objective of this study is to analyze the relationship between perceived emotional intelligence (PEI) and the emotional impact of cybervictimization. We hypothesize that EI, which has previously been found to play a role in traditional bullying and cyberbullying, may also affect the emotional impact of cyberbullying. The participants in our study were 636 university students from two universities in the south of Spain. Three self-report questionnaires were used: the “European Cyberbullying Intervention Project Questionnaire,” the “Cyberbullying Emotional Impact Scale”; and “Trait Meta-Mood Scale-24.” Structural Equation Models were used to test the relationships between the analyzed variables. The results support the idea that PEI, by way of a moderator effect, affects the relationship between cybervictimization and emotional impact. Taken together, cybervictimization and PEI explain much of the variance observed in the emotional impact in general and in the negative dimensions of that impact in particular. Attention and Repair were found to be inversely related to Annoyance and Dejection, and positively related to Invigoration. Clarity has the opposite pattern; a positive relationship with Annoyance and Dejection and an inverse relationship with Invigoration. Various hypothetical explanations of these patterns are discussed.

## Introduction

Cyberbullying has been defined as intentional, unjustified attacks carried out repeatedly using computers, cell phones, and other electronic devices from which victims cannot easily defend themselves (Patchin and Hinduja, 2012). According to the review conducted by Kowalski et al. (2014), most of the studies that have addressed this problem show that between 10 and 40% of secondary school pupils have been involved in cyberbullying, while other research suggests that as many as 72% have at some time experienced it (Juvonen and Gross, 2008). As all aggressive acts, and especially those sustained over a period of time, cyberbullying is or may be harmful to its victims. Cyberbullying negatively affects different areas of victims’ lives, above all their emotional balance and social adjustment. Cyberbullying victimization has been associated with negative emotions such as sadness, shame, guilt, loneliness, and helplessness (Ortega et al., 2009, 2012b; Sahin, 2012), psychosomatic problems (Carter, 2011; Beckman et al., 2012), depressive symptomatology (Perren et al., 2010; Olenik-Shemesh et al., 2012), anxiety symptomatology (Sontag et al., 2011), low self-esteem and having a negative self-concept (Didden et al., 2009), and with alcohol, tobacco, and drug use (Ybarra and Mitchell, 2004). Cybervictimization has even been related to an increased likelihood of self-harm (Kessel Schneider et al., 2012) and suicidal thoughts (Bonanno and Hymel, 2013). Considerable overlap has also been identified between cybervictimization and traditional victimization (Gradinger et al., 2009; Katzer et al., 2009; Del Rey et al., 2012).

However, the effects of cybervictimization are not found with the same degree of intensity in all victims (Ortega et al., 2012a; Dredge et al., 2014; McVie, 2014), and different cybervictim profiles have been identified based on the type of experienced emotions (Ortega et al., 2009, 2012b). Different theoretical models have been proposed to help understand the relationship between cyberbullying – and aggression in general – and its effects on victims (see Kowalski et al., 2014), most of which focus almost exclusively on cognitive variables (Lazarus and Folkman, 1984; Crick and Dodge, 1994; Anderson and Bushman, 2002). Nevertheless, sufficient empirical evidence exists to suggest that other kinds of variables are also important in determining the relationship between cybervictimization and its final impact. These variables relate to two dimensions: the aggressive behavior itself, including the type of cyberbullying (Smith et al., 2008; Ortega et al., 2009; Staude-Müller et al., 2009), its duration and severity (Dyer and Teggart, 2007; Aluede et al., 2008); and the personal features of victims of cyberbullying. Besides cognitive variables, the latter dimension includes social and emotional variables: social skills, which can alleviate or reduce the risk of developing depressive symptoms (Vassallo et al., 2014); coping strategies, which help victims play down the importance of the problem and its consequences (Perren et al., 2012); resilience, with resilient individuals showing less vulnerability and a greater capacity to recover from adversity (Ttofi et al., 2014); personality traits, some of which – such as the tendency to over-control – are linked to a higher probability of a greater impact (Overbeek et al., 2010); social intelligence, which has been found to be negatively related to traditional victimization and cybervictimization (Schultze-Krumbholz and Scheithauer, 2009; Hunt et al., 2012); and emotional intelligence (EI), with higher levels of EI being associated with a lower likelihood of being involved in cyberbullying (Elipe et al., 2012; Baroncelli and Ciucci, 2014). This study focuses on the last of these variables.

Emotional intelligence is a concept established in the 1990s by Salovey and Mayer (1990; Mayer and Salovey, 1997). It refers to those aspects of intelligence that relate to the management of one’s own emotions and those of others. In the model proposed by these authors, EI is composed of four branches: recognizing or perceiving emotions, i.e., the capacity to perceive emotions in oneself and others efficiently; using emotions to facilitate thinking; understanding emotions; and managing emotions. Mayer et al. (2008) found that those individuals who are better at perceiving, understanding, using and managing both their own emotions and those of others display higher levels of social adjustment. Other empirical studies have found correlations between these skills and different social and emotional adaptation strategies (for an overview see Extremera and Fernández-Berrocal, 2005; Fernández-Berrocal and Extremera, 2008).

A related concept is emotional metacognition, or perceived emotional intelligence (PEI), a term used to refer to an individuals’ perception of their own emotional skills. The most widely used instrument for measuring PEI is the Trait Meta-Mood Scale (TMMS; Salovey et al., 1995), which includes the following dimensions: Attention, defined as the perceived ability to focus on one’s own emotions; Clarity, defined as the perceived ability to understand one’s own emotional moods; and Repair, defined as the perceived ability to manage and control one’s own emotions. Studies carried out in this area have revealed the existence of a link between the different components of PEI and different aspects of emotional adjustment. More specifically, high scores in Clarity and Repair are inversely associated with depressive symptoms (Extremera and Fernández-Berrocal, 2006; Fernández-Berrocal et al., 2006), social anxiety (Salovey et al., 2002) and personality disorders (Leible and Snell, 2004), but positively associated with levels of well-being and life satisfaction (Extremera et al., 2011). It has also been shown that individuals with higher levels of psychological adaptation generally score low on Attention, and high on Clarity and emotional Repair (Extremera and Fernández-Berrocal, 2005). So, whereas high levels of Clarity and Repair are related to understanding and managing emotions, Attention is related to perceiving one’s own emotions, but too much of this without the accompaniment of good emotional management can lead to ruminative thought processes.

A number of studies have analyzed the relationship between aggressive behavior in general and EI, and most of them find significant empirical evidence to suggest a link does indeed exist. In a systematic review of the literature by García-Sancho et al. (2014) just over 94% of the listed studies found an inverse relationship between EI and aggressive behavior, regardless of the socio-cultural context of the studies, the age of the subjects in the samples or the type of aggression. Peláez-Fernández et al. (2014) revealed that PEI helps explain aggressive conduct over and above the effect of age, sex, and personality traits. In their study, the PEI dimensions moderate the relationship between aggression and personality. Research looking specifically at the relationship between EI and bullying has also found empirical evidence of the importance of this link (Oluyinka, 2009; Mavroveli and Sanchez-Ruiz, 2011; Kokkinos and Kipritsi, 2012). Several studies have explored the correlation in greater depth by looking at the different dimensions of EI separately. Lomas et al. (2012) showed that understanding others’ emotions is negatively related to the involvement in bullying, and that low scores in “emotion management and control” were linked to higher levels of self-reported victimization. Elipe et al. (2012) concluded that victims and bully-victims of traditional bullying are more likely to show higher levels of Attention and lower levels of Clarity, confirming results of an earlier study (Elipe et al., 2011), but they did not find these relationships to be significant in the case of cyberbullying. Other researchers have linked EI to certain dimensions of the dynamics of bullying and cyberbullying. Downey et al. (2010) found that people with lower scores in EI tend to use non-productive coping strategies, attempting to reduce their stress rather than to seek a solution to the conflict. Extremera and Fernández-Berrocal (2005) had previously argued that difficulty in identifying one’s own emotions, often linked to high scores in Attention, could mean a decrease in the cognitive resources dedicated to choosing efficient coping strategies, i.e., individuals need to understand what is happening to them and without this may find it harder to successfully address the problem. Taking into account that coping strategies are considered key elements in tackling bullying and cyberbullying (e.g., Hunter and Borg, 2006; Nabuzoka et al., 2009), understanding the relationship between EI and coping could be important.

Despite the considerable attention in the literature given to the relationship between EI and (cyber)bullying, it is still not fully understood, especially with regard to EI’s role in moderating the emotional impact. The aim of this study is precisely to make progress in this direction, and to learn more about how to counter or eliminate that impact.

Our principal objective is to analyze the role of PEI with regard to the emotional impact of cybervictimization. Our starting hypothesis is that PEI acts as a moderator between cybervictimization and emotional impact. More specifically, following earlier empirical evidence, we hypothesize the existence of a positive link between the level of focus on one’s own emotions and negative emotional impact, and an inverse link between understanding and, above all, management of emotions and negative emotional impact.

## Materials and Methods

### Participants

The participants were 638 undergraduates from the Humanities and Educational Sciences faculties of the University of Jaén (*n* = 328) and the University of Seville (*n* = 308), in the south of Spain. Two uncompleted questionnaires were discarded, and the final sample therefore comprised 636 students, 68.7% of whom were girls. The age range was 18–61, with 95% of the population being between 18 and 25 (*M* = 20.45, SD = 4.13). The students were enrolled in courses leading to qualifications in Teacher Training (*n* = 409), Psychology (*n* = 173) and Psychopedagogy (*n* = 54). The participation in the study was voluntary. Data were collected following the general principles and the ethical research standards of the American Psychological Association (APA).

### Procedure

After obtaining authorization from the teachers of the different courses, an informative talk was conducted with the students in which the objectives of the study were explained and the students were invited to collaborate. After informed consent, those interested completed a pencil and paper questionnaire during class time, which took ∼30 min. The voluntary nature of participating in the study was highlighted so that any student could leave the class at that time if they did not want to participate. In addition, the complete anonymity of the questionnaires was explained to participants, and a guarantee was given that the data would only be used for research purposes.

### Instruments

The instruments used to evaluate the variables under study were self-report questionnaires with Likert-type multiple-choice scales.

Cyberbullying was evaluated using the Spanish version of the “European Cyberbullying Intervention Project Questionnaire, ECIPQ” (Del Rey et al., in press). This questionnaire has 22 items covering cyberbullying in the 2 months prior to participation in the survey, with one subscale for cybervictimization (11 items) and another for cyber-aggression (11 items). Answers are entered on a scale of 1 to 5: 1 Never; 2 Once or twice; 3 Once or twice monthly; 4 About once a week; 5 More than once a week. The included forms of cyberconduct are: Insults said to me; Insults about me said to others; Threats; Identity theft; Use of personal identity without permission; Private information theft; Display of private information; Embarrassing videos or pictures; Manipulation of pictures; Social exclusion; and Spreading of rumors. This scale has displayed good psychometric properties in studies carried out to date (Ortega-Ruiz et al., 2012; Casas et al., 2013). However, since this study employed only the cybervictimization (CV) subscale, a confirmatory factor analysis (CFA) was used in order to test its appropriateness. The results indicated a good-fit for the measurement model, except for the Chi-square (due to its sensitivity to sample size): X^2^_S-B_ = 102.95; DF = 49; NNFI = 0.99, CFI = 0.99; GFI = 0.98; RMSEA = 0.038; SRMR = 0.047; ECVI = 0.78 (for more details about the analysis and the interpretation of indices, see the statistical analysis section). The internal consistency, Cronbach’s alpha, was 0.90.

Emotional impact was evaluated using the “Cyberbullying Emotional Impact, CBEI” scale (Elipe et al., unpublished). This scale is an adaptation of the PANAS scale specifically designed to analyze cyberbullying situations. It lists a series of emotions and asks subjects to grade the extent to which they would feel those emotions if they were a cybervictim on a scale of 1 to 5 (Not at all [1] to A lot [5]). The scale has three subscales for different types of impact: Invigoration (including Animated; Energetic, Lively; Satisfied, Proud; Determined, Daring; Active); Dejection (Tense, Distressed; Guilty; Lonely; Ashamed; Defenseless; Depressed; Worried; Scared); and Annoyance (Upset, Bothered; Angry, Annoyed; Irritable, In a bad mood). Since this scale had not been validated beforehand, a measurement model was estimated to test whether the observed items reliably reflected the latent variables. The results confirmed the proposed model’s goodness-of-fit: X^2^_S-B_ = 305.48; DF = 101; NNFI = 0.967, CFI = 0.97; GFI = 0.99; RMSEA = 0.057; SRMR = 0.059; ECVI = 2.26. Cronbach’s Alpha was 0.71 for the overall scale, 0.84 for Invigoration, 0.90 for Dejection, and 0.76 for Annoyance.

Perceived emotional intelligence was evaluated using the Spanish version of the “Trait Meta-Mood Scale-24” (Fernández-Berrocal et al., 2004), a scale comprising 24 items with which subjects are asked to express their degree of agreement on a Likert-type scale of 1 to 5 (Not at all [1] to Totally [5]). The scale, which has three subscales – Attention, Clarity, and Repair – has displayed good psychometric properties in earlier studies (Fernández-Berrocal et al., 2004), and the CFA confirmed its appropriateness for the sample in our study: X^2^_S-B_ = 1052.96; DF = 249; NNFI = 0.95, CFI = 0.96; GFI = 0.95; RMSEA = 0.071; SRMR = 0.074; ECVI = 1.82. In this case, Cronbach’s Alpha was 0.91 for the overall scale, 0.90 for Attention, 0.88 for Clarity, and 0.85 for Repair.

### Statistical Analysis

The proposed models were tested using structural equation methods. Taking into account the ordinal nature of the variables involved, robust methods were employed (Flora and Curran, 2004). Specifically, in those analyses which included the cybervictimization variable – the CFA of the cybervictimization scale and Models 1 and 3 – the unweighted least squares (ULSs) method was used to take into account deviations due to non-normally distributed variables. This was necessary because neither the normality nor the kurtosis conditions were satisfied (see **Table 1**). This method has proved to be one of the most accurate and reliable methods for estimating models with ordinal variables that do not fulfill normality conditions (Forero et al., 2009). In the other analyses – the CFA of the emotional impact and EI scales and Model 2, in which the included variables (PEI and emotional impact) did not significantly deviate from normality conditions – robust maximum likelihood (RML) was adopted as the most appropriate method (Hu and Bentler, 1998).

**Table 1:** Descriptive statistics and Spearman correlation for the variables included in the study.

	1	2	3	4	5	6	7
(1) CV	1						
(2) Invigoration	0.06	1					
(3) Dejection	0.01	-0.44^∗∗^	1				
(4) Annoyance	-0.05	-0.24^∗∗^	0.53^∗∗^	1			
(5) Attention	0.06	-0.07	0.25^∗∗^	0.16^∗∗^	1		
(6) Clarity	-0.21^∗∗^	0.11^∗^	-0.13^∗∗^	-0.05	0.13^∗∗^	1	
(7) Repair	-0.11^∗^	0.24^∗∗^	-0.21^∗∗^	-0.12^∗∗^	-0.01	0.41^∗∗^	1
*M* (range: 1–5)	1.14	1.72	3.03	3.64	3.44	3.47	3.49
SD	0.24	0.84	1.01	0.98	0.78	0.74	0.76
Skewness	2.99	1.43	-0.19	-0.54	-0.19	-0.14	-0.15
Kurtosis	11.94	1.63	-0.96	-0.34	-0.50	-0.39	-0.46

^∗^*p* < 0.01; ^∗∗^*p* ≤ 0.001.

Since this scale had not been validated previously, both exploratory factor analysis (EFA) and CFA were used to do so. The sample was randomly divided into two halves, using EFA to assess the factor structure from sample A (*n* = 298) and then using CFA to confirm the obtained factor structure using Sample B (*n* = 338).

To compare the suitability of the proposed models, we adhered to the recommendations by Hu and Bentler (1995, 1999), and combined different fit indices with the recommended cutoff values: Chi-square over degrees of freedom ratio with a recommended cutoff of <3; root mean square error of approximation (RMSEA) with recommended cutoff values <0.08; standardized root mean square residual (SRMR) <0.05; goodness of fit index (GFI); non-normed fit index (NNFI), and comparative fit index (CFI) with recommended values >0.90; and expected cross-validation index (ECVI), for which the smallest values indicate the greatest potential for replication (Browne and Cudeck, 1989).

Since missing data for the different variables did not exceed 2% (13 cases), and for most variables being lower than 1%, they were not imputed.

The analyses were performed using the SPSS 21 statistical package, and LISREL 9.1 (Jöreskog and Sörbom, 2012), a package that allows the estimation of polychoric correlations, which are best suited to the variables involved (Jöreskog, 1994).

## Results

**FIGURE 1:**
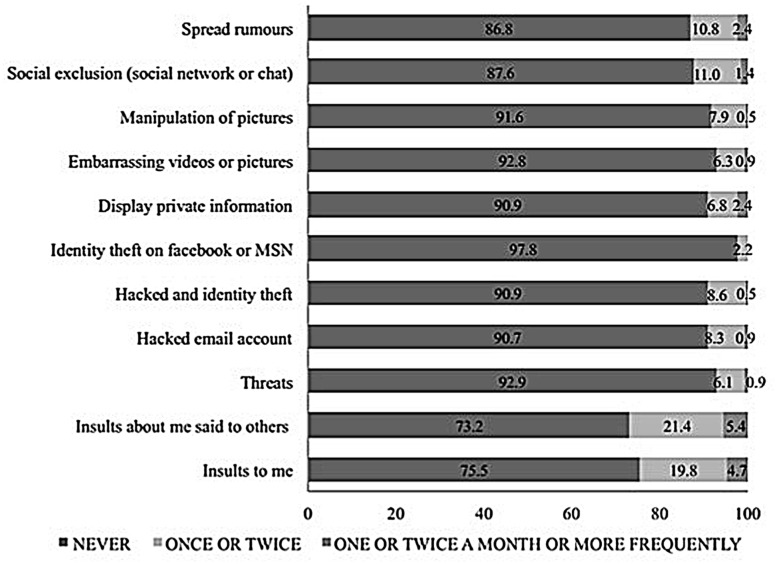
**Percentage of students who reported having experienced the different type of cybervictimization**.

Over half of the subjects (54%) reported having experienced one or more of the 11 listed types of cybervictimization in the past 2 months (**Figure 1**) . The most frequently experienced forms were insults about me said to others via internet or SMS messages, followed by direct personal insults via email or SMS messages (one out of every four pupils). Just over one in ten reported having been excluded or ignored in a social network or chat site and having been the subject of rumors spread via internet. Only 2.2% said that somebody had created a false Facebook or MSN account to steal their identity; in those cases this happened once or twice.

With regard to the distribution of the latent variables included in the study, **Table 1** shows the main univariate descriptive statistics and the correlation between the variables. It is interesting to note the existence of significant correlation between all the EI factors and the different emotional impact factors. Attention is positively related to the Dejection and Annoyance Impacts, while Repair is inversely related to those factors and positively related to Invigoration. Clarity was found to have a significant positive link with Invigoration and an inverse link with Dejection. There was also an inverse link between CV and Clarity, and CV and Repair.

### Structural Models

The correlations between the different constructs were analyzed using structural equation models. First, two simple models were created to analyze how CV and PEI correlated with emotional impact. A third model was then designed, incorporating both variables simultaneously.

**FIGURE 2:**
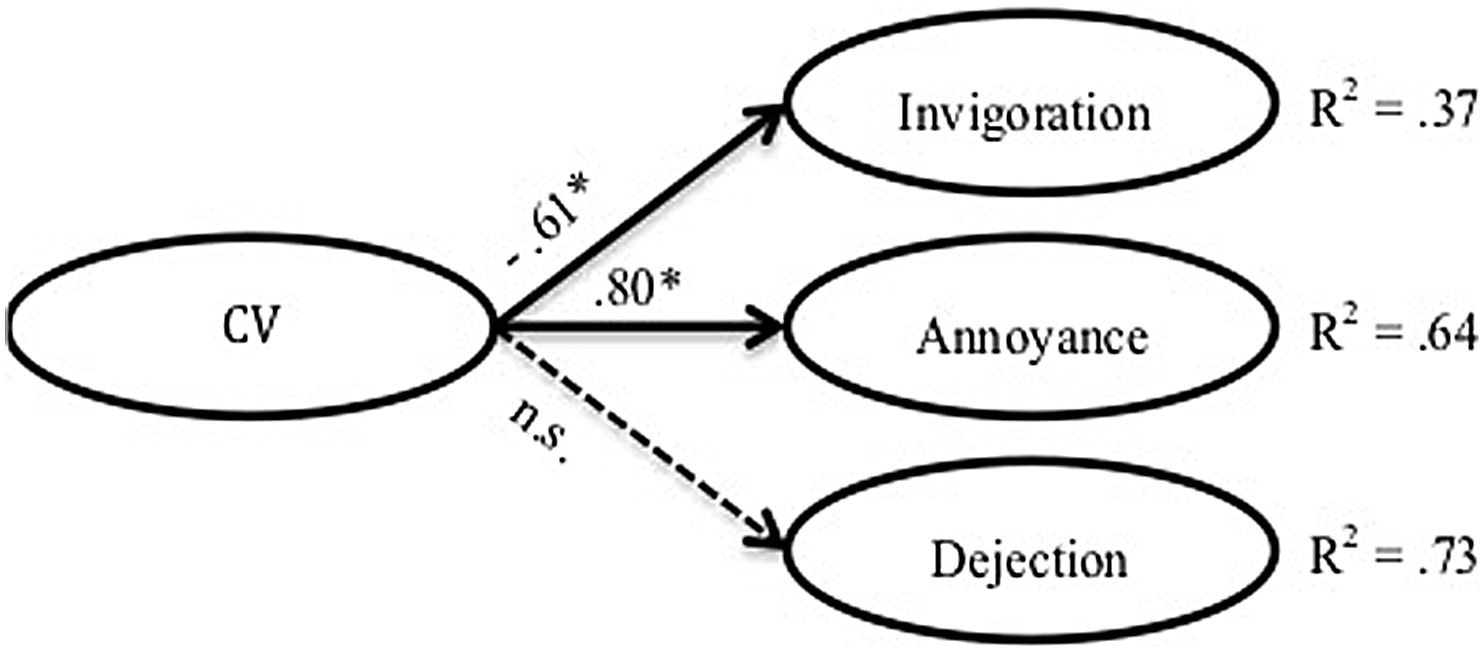
**Model of the direct link between CV and emotional impact. ^∗^*p* < 0.05. The discontinuous arrows indicate non-significant correlations**.

**FIGURE 3:**
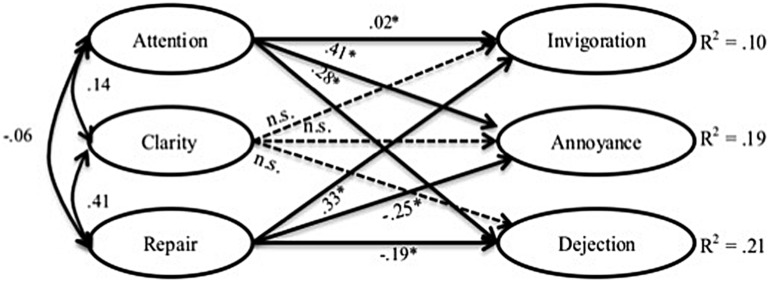
**Model of the direct link between perceived emotional intelligence and emotional impact.** ^∗^*p* < 0.05. The discontinuous arrows indicate non-significant correlations.

**FIGURE 4:**
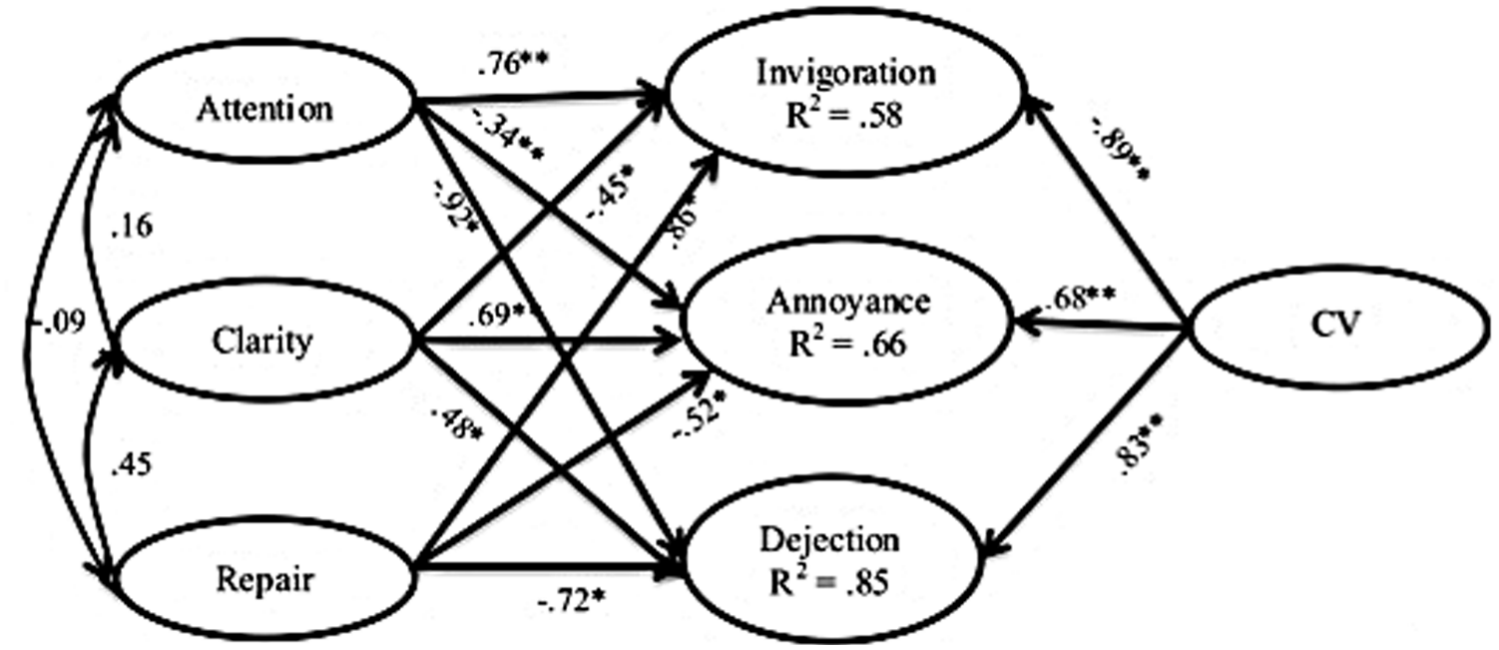
**Model of the links between cybervictimization, perceived emotional intelligence and emotional impact.** ^∗^*p* < 0.01; ^∗∗^*p* ≤ 0.005.

The fit indices of these models are shown in **Table 2**. **Figures 2, 3**, and **4** show the models themselves, including their standardized regression coefficients. For ease of viewing, observed items of the latent variables and error terms have both been omitted from the figures.

**Table 2:** Model fit indicators.

	X^2^_S-B_	*df*	GFI	NNFI	CFI	RMSEA	SRMR	ECVI
Model 1	1786.04	321	0.79	0.89	0.85	0.085 (0.081; 0.089)^a^	0.17	2.99
Model 2	1963.00	725	0.95	0.96	0.96	0.036 (0.030; 0.039)^a^	0.04	2.86
Model 3	3555.88	1202	0.98	0.95	0.95	0.042 (0.040; 0.044)^a^	0.09	5.98

^a^Confidence interval 90%.

As can be seen in **Table 2**, the fit of the first model (**Figure 2**), which describes the relationship between CV and emotional impact, is not satisfactory; most of the index values lie outside the proposed cut-off points.

In contrast, all fit indicators of Model 2 (**Figure 3**) lie within the commonly accepted cut-off points. However, as can be seen in the figure, the explained variance for each impact factor is rather low, not exceeding 21%. Whereas Attention has a significant positive correlation with the three impact factors, especially with Annoyance and Dejection, Repair correlates positively with Invigoration and inversely with Dejection and Annoyance. Clarity was found to have no significant correlations with emotional impact.

The third model (see **Figure 4**) produced better fit indices than the first two models. The explained variance of the included variables can also be considered satisfactory, rising as high as 85% for Dejection. Analysis of the beta coefficients showed that when the two constructs were included in the same model, significant correlation appears between all variables. Specifically, in the case of Attention, the simultaneous inclusion of CV considerably altered the correlations with emotional impact, leaving a significant positive link only with Invigoration and changing the earlier positive links with Annoyance and Dejection into inverse ones. Correlations between Clarity and emotional impact became significant, showing an inverse link with Invigoration (that is to say, the greater the clarity the lower the impact) and positive links with Annoyance and Dejection. Repair displayed the same correlations as in the earlier model – a positive link with Invigoration and inverse links with Annoyance and Dejection – although the magnitude of those correlations increased considerably. CV was found to correlate significantly with all three types of impact, inversely in the case of Invigoration and positively in the case of the other two.

## Discussion

The results obtained in this study show that cyberbullying is a problem, albeit not an excessively serious one, among university students. Over half the subjects in the sample reported to have experienced some type of cybervictimization in the 2 months prior to the survey. For two reasons, this prevalence rate is difficult to compare to those found in other studies: (a) major conceptual and methodological differences can cause substantial variation in prevalence rates; and (b) very few studies investigating this phenomenon have used samples drawn from university populations. In fact, of the 131 studies in the meta-analysis conducted by Kowalski et al. (2014), only eight had samples made up of university students.

The most common forms of cyberbullying found in this study are defamation, insults and exclusion from social networks and/or chat or messaging groups. Defamation and insults were also found to be the most frequent types of conduct in earlier studies on cyberbullying (Katzer and Fetchenhauer, 2007; Staude-Müller et al., 2009). Moreover, these three types of behavior – insults, defamation and social exclusion – also constitute the most frequent forms of conduct found in studies into traditional bullying (e.g., Nansel et al., 2001; Díaz-Aguado et al., 2010; Lemstra et al., 2011). This supports the idea that cyberbullying, or part of cyberbullying, should be understood as a variation or an indirect form of traditional bullying (Ortega and Mora-Merchán, 2008; Olweus, 2013; Ortega-Ruiz et al., 2014). Several studies have shown how cyberbullying, and more specifically cybervictimization, occur as the result of, and can be predicted by, traditional victimization, although this relationship is not seen in the other direction (Del Rey et al., 2012; Hemphill et al., 2012; Kowalski et al., 2012; Olweus, 2012).

The principal objective of this study was to analyze the role of PEI with regard to the emotional impact of cybervictimization. The first interesting discovery was that CV and PEI have no clear, significant link with emotional impact when the two variables are analyzed separately. Models created to explore these links resulted in a poor model fit in the case of CV and in low proportions of explained variance in the case of PEI. However, including both variables together improved the model fit, and led to a considerably higher proportion of variance explained for each emotional impact factor. These results appear to confirm our starting hypothesis, that PEI acts as a moderator variable between cybervictimization and emotional impact, attenuating or increasing the different dimensions of emotional impact – Invigoration, Annoyance, and Dejection – depending on the factor being considered.

Contrary to what might have been expected, CV was found to have no direct relationship with emotional impact. This was indicated both by the absence of any significant correlations and by the poor fit of model 1. These results seem to contradict studies that have identified links between frequency of harassment and emotional impact of traditional bullying (Dyer and Teggart, 2007; Aluede et al., 2008). The explanation may lie in their use of repetition as a defining criterion of cyberbullying. Due to the nature of ICT, one single episode of cyberbullying can live on in time and/or may be witnessed by a very large audience (Vandebosch and Van Cleemput, 2008; Menesini et al., 2012). Therefore, in the case of CV, the emotional impact may not depend on frequency. However, when PEI is included in the analysis, the results become very different and reveal that CV and emotional impacts are linked. This suggests the existence not of a direct link but of an indirect one, which is moderated by PEI. As Dredge et al. (2014) pointed out when discussing the varying impact of cyberbullying on victims, it is necessary to identify which variables affect and/or moderate the correlation. Without these variables it is impossible to gain a full understanding of the relationship.

The same applies to the link between PEI and emotional impact. Although in this study we found a model with good fit indices, the low proportion of variance explained shows that, when analyzed separately, PEI cannot sufficiently explain the emotional response to cyberbullying (at best it explained 21% of the variance of the Dejection response). However, PEI becomes much more important when it is included in the model alongside CV, explaining up to 85% of the variance of the Dejection response, and also reaching high levels for the two other categories of emotional impact. This highlights the importance of meeting the challenge to understand the true weight of the emotional variables. That is to say, it is only when the needs or the specific problem at hand – in this case CV – are taken into account that emotional skills take on importance as an aid in understanding the impact. When considered in an abstract manner they do not produce the same results. This appears to concur with findings in certain coping strategy analyses, which suggest it is not possible to evaluate the effectiveness of coping responses abstractly, because they are only effective when linked to a specific result (Somerfield and McCrae, 2000).

With regard to the relationship between the specific dimensions of PEI and emotional impact, the results in part support our hypotheses and in part contradict what we expected. The results of the second model confirm the proposed hypothesis that there exists an inverse relationship between Repair and negative emotional impact – Annoyance and Dejection – and a positive relationship between Attention and these two responses. These findings also corroborate the results obtained in earlier studies, in which comparative profiles between PEI and different emotional adjustment indicators follow the same pattern (Extremera and Fernández-Berrocal, 2005; Fernández-Berrocal and Extremera, 2008; Elipe et al., 2011; Ortega et al., 2011; Lomas et al., 2012; Peláez-Fernández et al., 2014).

When PEI is considered together with CV, however, the results are more difficult to interpret, especially those pertaining to the Attention and Clarity dimensions. Contrary to our expectations, the Attention variable had the same profile as the Repair variable – a positive link with Invigoration and an inverse link with Annoyance and Dejection – while Clarity showed just the opposite – an inverse link with Invigoration and a positive link with Annoyance and Dejection. It therefore appears that in cybervictimization high levels of Attention together with high levels of Repair tend to reduce both anger and depression-related manifestations of negative emotional impact, while at the same time facilitating a more dynamic response, which would presumably trigger more effective coping strategies. In contrast, Clarity seems to work the other way round, which leads us to think that a high level of Clarity when unaccompanied by an ability to change emotions cannot abate the negative emotional impact of CV and merely makes individuals more aware of the discomfort they are experiencing. In other words, knowing how you feel but do not knowing how to handle these feelings is not helpful in adapting to the situation. It is possible that our results are influenced by variables not considered in this study. As Peláez-Fernández et al. (2014) found in their study, PEI dimensions interact with personality variables in such a manner that, generally speaking, a high level of Attention tends to maximize the emotional experience and prolong negative moods, especially when accompanied by low levels of Clarity and Repair but this may vary with personality type. The above mentioned study also showed that understanding one’s own emotional state may contribute to an increase in anger, especially in provocation scenarios.

Once PEI was included in the model, the relationship between CV and emotional impact was as expected: CV had a positive link with the two negative emotional dimensions – Annoyance and Dejection – and an inverse link with Invigoration. This result supports the findings of earlier studies into cyberbullying, in which the most prevalent emotions among victims were, firstly, those associated with anger, such as anger and upset, followed by range of other negative emotions, such as helplessness, fear and worry (Katzer and Fetchenhauer, 2007; Ortega et al., 2009). The inverse relationship with Invigoration may indicate that it is those students who have not experienced cyberbullying or have experienced it very infrequently, who detect and report these types of emotions. If true, it could indicate the existence of differences between perceptions prior to experiencing the problem and perceptions once the problem is being experienced. Exploration of this possibility is beyond the scope of this study, but constitutes a possible avenue for future research.

Summarizing the obtained results, it can be concluded that PEI is clearly a variable which affects emotional impact, although its importance mainly emerges when considered in the context of a specific cause of that emotional impact, such a CV. Our findings suggest that strengthening emotional skills, especially emotional repair, could be an interesting addition to the traditionally used variables (e.g., improving social skills or giving information about ICT) in programs to prevent cyberbullying or to minimize its negative consequences.

### Strengths, Limitations, and Future Research

This study has a number of strengths and limitations worth mentioning. The first strength to highlight is its analysis of emotional impact. Numerous earlier studies have focused on the “most devastating” consequences of cybervictimization, such as depression and personality disorders, but few have proposed any way of measuring its specific, “immediate” emotional impact. Moreover, as far as we know, no other study on this theme has ever included a “non-negative” dimension of emotional impact such as Invigoration. Although it may seem logical to assume that all the emotional consequences of cybervictimization are emphatically negative, the fact that some students, albeit very few, reported non-negative responses may suggest that this assumption is a distortion of reality. Another strong point of the study is its sample of university students. As mentioned earlier, very few studies to date have analyzed cyberbullying in this layer of the population. Our results reveal the need for further research within the university population, given the confirmed presence of cyberbullying therein.

The study’s limitations mainly have to do with its sample and design. Although, as mentioned above, the sample is one of the strengths of this research project, it would nevertheless be interesting to extend it to subjects from lower education levels – secondary and high school pupils – to make evolutionary comparisons. In addition, a bigger sample would have allowed us to study the effect of gender and age on the relationship between our variables with enough statistical and methodological rigor. Furthermore, the study’s transversal design limited interpretation with regard to the directionality of the found relationships. A more dynamic approach, using longitudinal data, would make it possible to monitor the way cybervictimization evolves in subjects with specific emotional profiles, and this would contribute much toward our understanding of the phenomenon. It would reveal, for example, whether impact profiles remain the same or alter in the same person depending on the length of time they are ensnared in cyberbullying.

While providing answers to some questions, this study has also opened up avenues to future research. One of the first issues to emerge in the analysis was whether impact profiles were linked to specific courses of action or coping strategies. Key for understanding this aspect is to clarify the sequence of phases from initial subjection to cyberbullying, its emotional impact, to the conduct displayed by the victim, and to determine how the situation changes depending on the impact and the victim’s reaction. It is important to unravel the dynamic entanglement of actions and responses which shape the process of cybervictimization. Besides, it should be pointed out that although this study has focused exclusively on cybervictims, sufficient empirical evidence exists to show that emotional and behavioral problems are also experienced by perpetrators (Leiner et al., 2014). In view of the practical implications, specific analysis of the role played by PEI with regard to students’ different forms of involvement – as cybervictims, cyberbully-victims or cyberbullies – may also improve our understanding of the phenomenon and suggest courses of action to help the different parties.

Finally, it would be useful to replicate the study using instruments capable of evaluating the phenomena from other perspectives. In the case of EI, it would be interesting to compare the results found here with those obtained using an ability-based EI test such as the MSCEIT (Brackett and Salovey, 2006).

## Conflict of Interest Statement

The authors declare that the research was conducted in the absence of any commercial or financial relationships that could be construed as a potential conflict of interest.
